# Indigenous device for in circuit delivery of bronchodilator drugs through MDI

**DOI:** 10.4103/0019-5049.65353

**Published:** 2010

**Authors:** Balkar Singh, Nishkarsh Gupta, Bishnu Prasad Panigrahi, Deep Arora, Pradip Govil, Shibani Das, Manish Singh, Raj Tobin

**Affiliations:** Department of Anesthesia and Pain Medicine, Max Superspeciality Hospital, Saket, New Delhi, India

Sir,

Nebulized bronchodilator drugs are commonly used in mechanically ventilated patients but are expensive,[1] provide a possible source of contamination[2] and require adjustments in minute ventilation during delivery.[3]

In contrast drugs administration by MDI is easier, faster and provides cost-effective drug delivery. But the direct delivery of the drug into the circuit with MDI is difficult and may be inefficient.

Syringe actuated MDI have been described in past but they may be associated with loss of drug because of impaction on the syringe and catheter walls and mucosal injury due to impact of propellant on the tracheal mucosa.[4]

**Figure 1:**
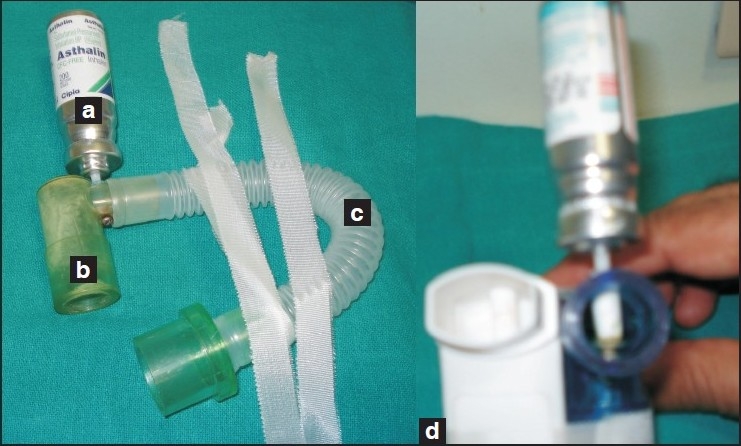
Indigenous device for in circuit delivery of bronchodilator drugs through MDI. (a) Metered dose inhaler (b) Right angle connector (c) Corrugated catheter mount (d) Nozzle of MDI

We describe a simple, indigenous, cheap device which can be used to deliver bronchodilator drugs to the tracheobronchial tree in intubated patients in circuit without leaks [Figure 1]. The nozzle of MDI is removed, smoothened and drilled into a standard right angle connector and fixed with a screw. This assembly is then sterilized before use. The distal end of this angle connector is attached to corrugated catheter mount for flexibility and ease of use. The patient end can be connected to the ETT when required.

This device is easy to use and saves times so, can be handy in emergency situations. We are keeping this device on our emergency trolley and can deliver the bronchodilator drugs to the patients in few seconds with simple sequence pick - attach and deliver.

This device has been successfully used by us - on many occasions to save patients life intraoperatively and in intubated ICU patients.
